# Low serum 25-hydroxyvitamin D is associated with higher risk of frequent headache in middle-aged and older men

**DOI:** 10.1038/srep39697

**Published:** 2017-01-03

**Authors:** Jyrki K. Virtanen, Rashid Giniatullin, Pekka Mäntyselkä, Sari Voutilainen, Tarja Nurmi, Jaakko Mursu, Jussi Kauhanen, Tomi-Pekka Tuomainen

**Affiliations:** 1 Institute of Public Health and Clinical Nutrition, University of Eastern Finland, Kuopio, Finland; 2 Department of Neurobiology, A. I. Virtanen Institute, University of Eastern Finland, Kuopio, Finland; 3 Laboratory of Neurobiology, Kazan Federal University, Kazan, Russia; 4 Primary Health Care Unit, Kuopio University Hospital, Finland

## Abstract

Vitamin D has been suggested to have a role in various neurovascular diseases, but the data regarding headache is inconclusive. Our aim was to investigate the associations between serum 25-hydroxyvitamin D [25(OH)D], a marker for vitamin D status, and risk of frequent headache. The study population consisted of 2601 men from the population-based Kuopio Ischaemic Heart Disease Risk Factor Study (KIHD) from eastern Finland, aged 42–60 years in 1984–1989. The cross-sectional associations with prevalence of self-reported frequent headache (defined as weekly or daily headaches) were estimated with multivariable-adjusted odds ratios. The average serum 25(OH) concentration was 43.4 nmol/L (SD 18.9, min-max 7.8–136.1 nmol/L). A total of 250 men (9.6%) reported frequent headache. The average serum 25(OH)D concentration among those with frequent headache was 38.3 nmol/L (SD 18.8) and 43.9 nmol/L (SD 18.9) among those without frequent headache, after adjustment for age and year and month of blood draw (*P* for difference <0.001). After multivariable adjustments, those in the lowest vs. the highest serum 25(OH)D quartile had 113% (95% CI 42, 218%; *P* for trend <0.001) higher odds for frequent headache. In conclusion, low serum 25(OH)D concentration was associated with markedly higher risk of frequent headache in men.

Primary headaches, including migraine, are among the leading health problems and causes of disability in the modern working population^1^. Currently, there is a global trend in chronification of migraine and a growing number of cases of medication overuse headache due to improper use and/or overuse of painkillers^2^,^3^. Interestingly, medication overuse headache could develop not only due to overuse of opioids but even after ‘specific’ anti-migraine treatments with triptans^4^. Intractable forms of migraine resistant to specific anti-migraine treatments and ‘epidemics of medication overuse headache’ are raising an issue of more complex approaches on headache cure, including nutritional approach.

The role of vitamin D is widely discussed recently as the key dietary factor determining the likelihood of various neurovascular diseases^5^,^6^. This issue is of key importance to Nordic countries, including Finland, Sweden, Norway, Denmark, and other areas with limited year-round UVB exposure from the sun. Headache prevalence has been suggested to be related to increasing latitude and possibly to be less prevalent during summer^7^, suggesting a possible role for vitamin D exposure. However, there is little data regarding the association between vitamin D exposure and headache. In a small case-control study, vitamin D receptor polymorphisms were found to be more prevalent in migraine patients than in controls and be related to severity of headache^8^. In a cross-sectional study from Norway, non-smoking participants with migraine or non-migraine headache had lower serum 25(OH)D concentration^9^, whereas no difference was observed among smokers. Low serum 25(OH)D concentration was also associated with slightly higher odds for non-migraine headache, but not for migraine^9^. In contrast, in another case-control study, serum 25(OH)D concentration did not differ between controls and migraine patients^10^. In another study from Norway, headache was more prevalent among those with serum 25(OH)D <50 nmol/L^11^.

In three case-reports, supplementation with vitamin D and calcium reduced migraine attacks^12^,^13^,^14^. However, results from larger, randomized, placebo-controlled vitamin D supplementation trials have been mixed^15^,^16^. Recently, combined therapy with simvastatin and vitamin D_3_ reduced the number of days with migraine^17^. This finding supported the previous cross-sectional observation from the same study group that statin use was associated with lower odds for severe headache or migraine only among those with higher serum 25(OH)D concentrations^18^.

Overall, the study findings are inconsistent and many studies are small in size. Therefore, the purpose of our study was to investigate the associations between serum 25(OH)D and risk of frequent headache among 2601 middle–aged and older men from a population-based cohort study from eastern Finland.

## Methods

### Study Population

The Kuopio Ischaemic Heart Disease Risk Factor Study (KIHD) was designed to investigate risk factors for cardiovascular disease, atherosclerosis, and related outcomes in a population-based, randomly selected sample of men from Eastern Finland^19^. The baseline examinations were carried out in 1984–1989 for a total of 2682 men who were 42, 48, 54 or 60 years old (83% of those eligible). The baseline characteristics of the entire study population have been described previously^19^. The KIHD study protocol was approved by the Research Ethics Committee of the University of Kuopio. All subjects gave written informed consent for participation. All methods were performed in accordance with approved guidelines and regulations. Subjects with missing data on serum 25(OH)D concentration (n = 53) or headache (n = 28) were excluded, leaving 2601 men for the analyses.

### Measurements

Fasting venous blood samples were collected between 8AM and 10AM at the baseline examinations. Subjects were instructed to abstain from ingesting alcohol for three days and from smoking and eating for 12 hours prior to giving the sample. Detailed descriptions of the assessment of medical history and medications^20^, smoking^20^, alcohol consumption^20^, blood pressure^20^, and physical activity^21^ at baseline have been published. Education was assessed in years by using self-administered questionnaire. Annual income was obtained from a self-administered questionnaire. Hypertension diagnosis was defined as systolic/diastolic blood pressure >140/90 mmHg or use of hypertension medication. Body mass index (BMI) was computed as the ratio of weight in kilograms to the square of height in meters. Serum 25(OH)D_3_ was measured after hexane extraction with high-performance liquid chromatography using coulometric electrode array detection, as previously described^22^.

### Diagnostic Criteria for Frequent Headache

Headache diagnosis was based on the response to the question in the study questionnaire: “Have you had headache during the previous 12 months?” The choices were “not at all”, “less than once per month”, “monthly”, “weekly” and “daily”. In the current study, those with weekly or daily headache were classified as having frequent headache.

### Statistical Analysis

The univariate relationships between serum 25(OH)D and characteristics of the study population were assessed by means and linear regression (for continuous variables) or χ^2^-tests (for categorical variables). The mean serum 25(OH)D concentration in the subjects with frequent headache and in the rest of the study population were analyzed using analysis of covariance (ANCOVA), with adjustments for potential confounders. Multivariate-adjusted logistic regression was used to estimate odds ratios (OR) in quartiles of serum 25(OH)D, with the highest category as the reference. The model 1 included age (years), examination year and examination month. The Model 2 included the variables in the Model 1 plus body mass index (kg/m^2^), systolic and diastolic blood pressure (mmHg), smoking (never smoker, previous smoker, current smoker <20 cigarettes/d and current smoker ≥20 cigarettes/d), education years, income (euros), leisure-time physical activity (kcal/d), self-reported health (extremely good, quite good, average, quite poor, extremely poor), use of analgesics (drugs for headache, back pain, joint pain or other pain, opioids, or acetylsalicylic acid), and alcohol intake (g/d). All quantitative variables were entered in the models as continuous variables. Further adjustments for disease history (coronary heart disease, stroke, cancer, type 2 diabetes), number of concurrent medications used, or use of cardiovascular disease drugs that could potentially be associated with headache (beta-blockers, calcium channel blockers, angiotensin-converting-enzyme inhibitors) did not change the associations (<5% change in estimates). Cohort mean was used to replace missing values in covariates (<2.3%). Tests of linear trend were conducted by assigning the median values for each category of exposure variable and treating those as a single continuous variable. All *P*-values were 2-tailed (α = 0.05). Data were analysed using SPSS 21.0 for Windows (Armonk, NY: IBM Corp.).

### Data availability

The KIHD data is not publicly available.

## Results

### Population Characteristics

The characteristics of the study population according to the serum 25(OH)D quartile are shown in the Table 1. Those with a lower serum 25(OH)D concentration were more likely to be younger, have lower leisure-time physical activity, lower income and lower self-reported health. They were also more likely to be smokers.

In the whole study population, the average serum 25(OH)D concentration was 43.4 nmol/L (SD 18.9, min-max 7.8–136.1 nmol/L). Among the subjects, 67.9% had the serum 25(OH)D concentration <50 nmol/L and 6.9% had concentration ≥75 nmol/L. The average serum 25(OH)D concentration among those with frequent headache was 38.3 nmol/L (SD 18.8) and 43.9 nmol/L (SD 18.9) among those without frequent headache, after adjustment for age and year and month of blood draw (p for difference <0.001).

The proportion of subjects with frequent headache was lower among those men whose blood sample was drawn during the high UVB exposure months, i.e. between June and September (45 out of 626 men, 7.2%) than among the other men (205 out of 1975 men, 10.4%) (p for difference = 0.018).

### Associations of serum 25(OH)D with risk of frequent headache

Among the 2601 men, 250 men (9.6%) reported frequent headache. After adjustment for age and year and month of blood draw, those in the lowest serum 25(OH)D quartile had 116% higher odds (OR 2.16; 95% CI 1.49–3.13) for frequent headache (Model 1, Table 2). Further adjustments for potential confounders did not appreciably change the associations (Model 2, Table 2). However, although the p-value for trend across quartiles was statistically significant, the increased odds for frequent headache was present only in the lowest serum 25(OH)D quartile.

## Discussion

In this cross-sectional population-based cohort study among middle-aged men from eastern Finland, low serum 25(OH)D concentration was associated with a higher risk of frequent headache.

The role of vitamin D in headaches remains unclear, mainly due to predominantly small studies with inconsistent findings^8^,^9^,^10^,^11^,^12^,^13^,^14^,^15^,^16^,^17^,^18^ and the lack of large randomized supplementation trials that would show benefit after improvement of vitamin D status with vitamin D supplementation^23^. Our study, being one of the largest studies that have investigated the issue, supports the view that vitamin D may be beneficial in headache prevention. Besides the size of the study, the strengths of our study include its population-based recruitment and the extensive examinations for potential confounding factors.

One interesting issue is that how vitamin D can reduce the occurrence of headaches? The classical view suggests that the main signalling effect of vitamin D is mediated by its active metabolite 1,25(OH)_2_D via the regulation of transcription^24^. The regulation of transcription could include expression of ‘protective genes’, such as the anti-inflammatory cytokine IL-10^25^ or suppression of pro-inflammatory or pro-nociceptive genes, such as IL-1beta, TNF-alpha and others^23^. Vitamin D receptor (VDR) and the vitamin D metabolizing enzymes are expressed in nociceptive sensory neurons^26^, consistent with pro-nociceptive signalling in deficiency of vitamin D^27^. Vitamin D deficiency has also been associated with chronic tension-type headache, perhaps by causing musculoskeletal pain^28^,^29^.

Recently, activation of various transcriptional factors was linked also with fast non-genomic effects within the nociceptive system^30^. This is likely applicable also to signalling initiated by the vitamin D^25^. Thus, a rapid inhibitory effect of 1,25(OH)_2_D on ATP-gated P2X7 in human immune cells was reported^31^. Notably, P2X7 receptors expressed by almost all types of immune cells, including brain-residing microglial cells, are triggering the pro-inflammatory and pro-nociceptive cascades leading to chronification of pain^32^.

A major limitation of the current study is the cross-sectional design, so we could not assess temporal relationship or the direction of the association. For example, those who suffer from frequent headaches may be less likely to spend time outdoors and would thus be less exposed to the UVB light. However, this may not be that relevant in Finland and in other regions where UVB exposure is strong enough for vitamin D formation in the skin only during few months of a year. Also, despite extensive adjustment for potential confounders, we cannot fully exclude the possibility of residual confounding. Another limitation is the small proportion of subjects with higher serum 25(OH)D concentrations. The risk of headache may have been stronger, if the contrast in the serum 25(OH)D concentrations were larger. Our study also included only middle-aged men, so the findings may not be generalizable to other age-groups or to women. Finally, the outcome definitions were based on self-report, and we did not have information on the type of headache.

In conclusion, the results of this study in a Finnish population suggests an association between the serum 25(OH)D and risk of frequent headache. Large randomized vitamin D supplementation trials are needed to elucidate the role of vitamin D supplementation as a prophylaxis or treatment for headache.

## Additional Information

**How to cite this article**: Virtanen, J. K. *et al*. Low serum 25-hydroxyvitamin D is associated with higher risk of frequent headache in middle-aged and older men. *Sci. Rep.* **7**, 39697; doi: 10.1038/srep39697 (2017).

**Publisher's note:** Springer Nature remains neutral with regard to jurisdictional claims in published maps and institutional affiliations.

## Figures and Tables

**Table 1:** Characteristics of the 2601 participants according to serum 25-hydroxyvitamin D concentration.

Characteristic	Serum 25-hydroxyvitamin D quartile (nmol/L)				p for trend
	1 (<28.9)	2 (28.9–40.1)	3 (40.2–55.0)	4 (>55.0)	
Number of subjects	650	650	651	650	
Age (years)	52.8 (5.0)^a^	52.9 (5.1)	53.0 (5.3)	53.5 (5.0)	0.012
Body mass index (kg/m^2^)	26.7 (3.7)	26.9 (3.6)	27.0 (3.6)	26.8 (3.4)	0.80
Leisure-time physical activity (kcal/day)	115 (157)	130 (157)	154 (198)	167 (181)	<0.001
Income (euros)	11921 (7892)	13114 (8910)	13870 (9040)	13645 (9367)	0.001
Education (years)	8.5 (3.3)	8.8 (3.5)	8.7 (3.4)	8.7 (3.5)	0.57
Systolic blood pressure (mmHg)	135 (18)	134 (17)	134 (17)	134 (17)	0.17
Diastolic blood pressure (mmHg)	89 (11)	89 (11)	88 (10)	88 (10)	0.029
Alcohol intake (g/week)	73 (132)	77 (119)	77 (154)	75 (141)	0.92
Number of hangovers per year	4.1 (10.2)	4.0 (9.7)	3.8 (9.8)	4.1 (10.6)	0.96
Number of concurrent drugs	1.3 (1.8)	1.1 (1.6)	1.2 (1.6)	1.4 (1.8)	0.14
Use of analgesics (%)	17	16	16	20	0.24
Use of cardiovascular medications (%)	26	22	24	24	0.48
Self-reported health good/excellent (%)	33	35	37	40	0.005
Current smoker (%)	38	32	30	27	<0.001
Hypertension (%)	62	60	60	61	0.77
Coronary heart disease (%)	25	23	28	24	0.66
Stroke (%)	4	2	3	2	0.13
Cancer (%)	2	1	2	3	0.088
Type 2 diabetes (%)	7	6	7	4	0.081

^a^All values are means (SD) or percentages.

**Table 2:** Odds ratios (95% confidence intervals) of self-reported frequent headache in 2601 men according to serum 25-hydroxyvitamin D concentration.

	Serum 25-hydroxyvitamin D quartile (nmol/L)				p for trend
	1 (<28.9)	2 (28.9–40.1)	3 (40.2–55.0)	4 (>55.0)	
Cases/participants	98/650 (15.1%)	52/650 (8.0%)	48/651 (7.4%)	52/650 (8.0%)	
Model 1	2.16 (1.49–3.13)	1.03 (0.69–1.55)	0.93 (0.62–1.41)	1	<0.001
Model 2	2.13 (1.42–3.18)	1.02 (0.66–1.58)	0.87 (0.56–1.35)	1	<0.001

Model 1 is adjusted for age, examination year and month of blood draw.

Model 2 is adjusted for Model 1 plus body mass index (kg/m^2^), systolic and diastolic blood pressure (mmHg), smoking (never smoker, previous smoker, current smoker <20 cigarettes/d and current smoker ≥20 cigarettes/d), education years, income (euros), leisure-time physical activity (kcal/d), self-reported health (extremely good, quite good, average, quite poor, extremely poor), use of analgesics (drugs for headache, back pain, joint pain or other pain, opioids, or acetylsalicylic acid), and alcohol intake (g/d).
